# Supplementary material for: Single-Cell Transcriptomic Profiling of the Mouse Testicular Germ Cells Reveals Important Role of Phosphorylated GRTH/DDX25 in Round Spermatid Differentiation and Acrosome Biogenesis during Spermiogenesis
Source: Int J Mol Sci. 2023 Feb 4;24(4):3127. doi: 10.3390/ijms24043127 (PMC9962311; doi:10.3390/ijms24043127)
Supplement: Supplementary file 1 [file ijms-24-03127-s001.zip › Supplementary Figures.pdf]

Supplementary Figure S1

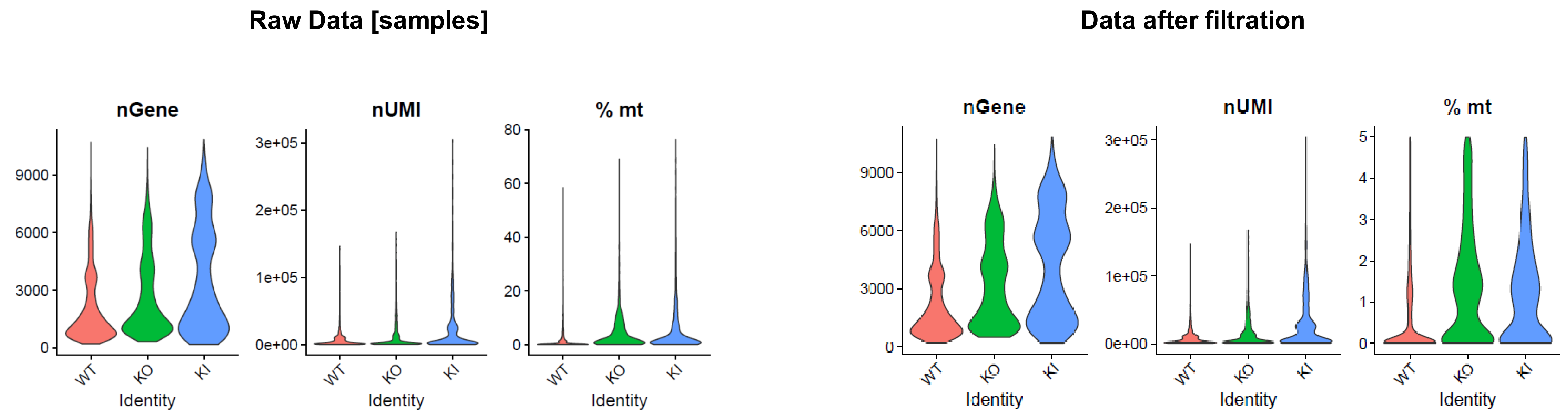

Supplementary Figure S2

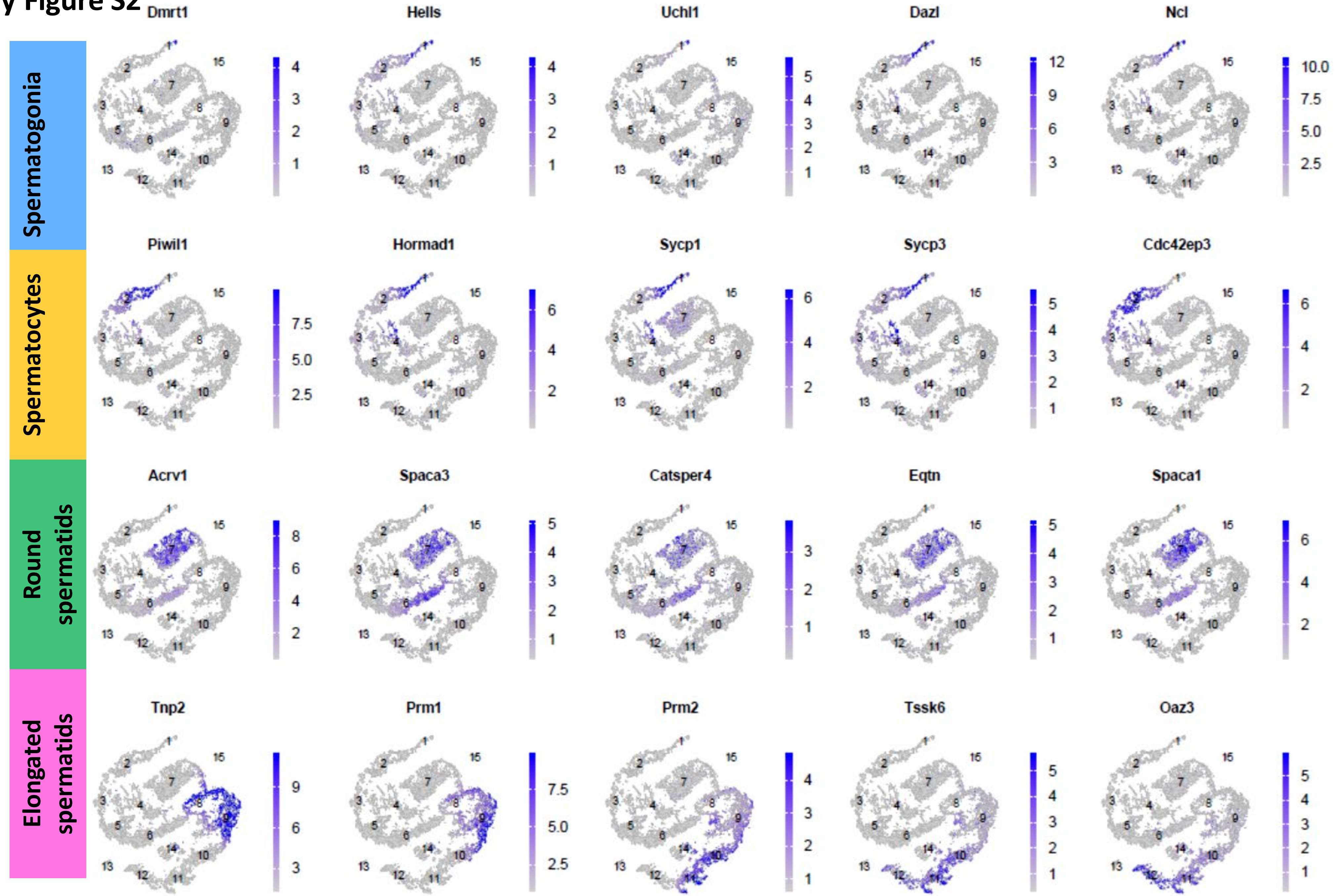

Figure S3

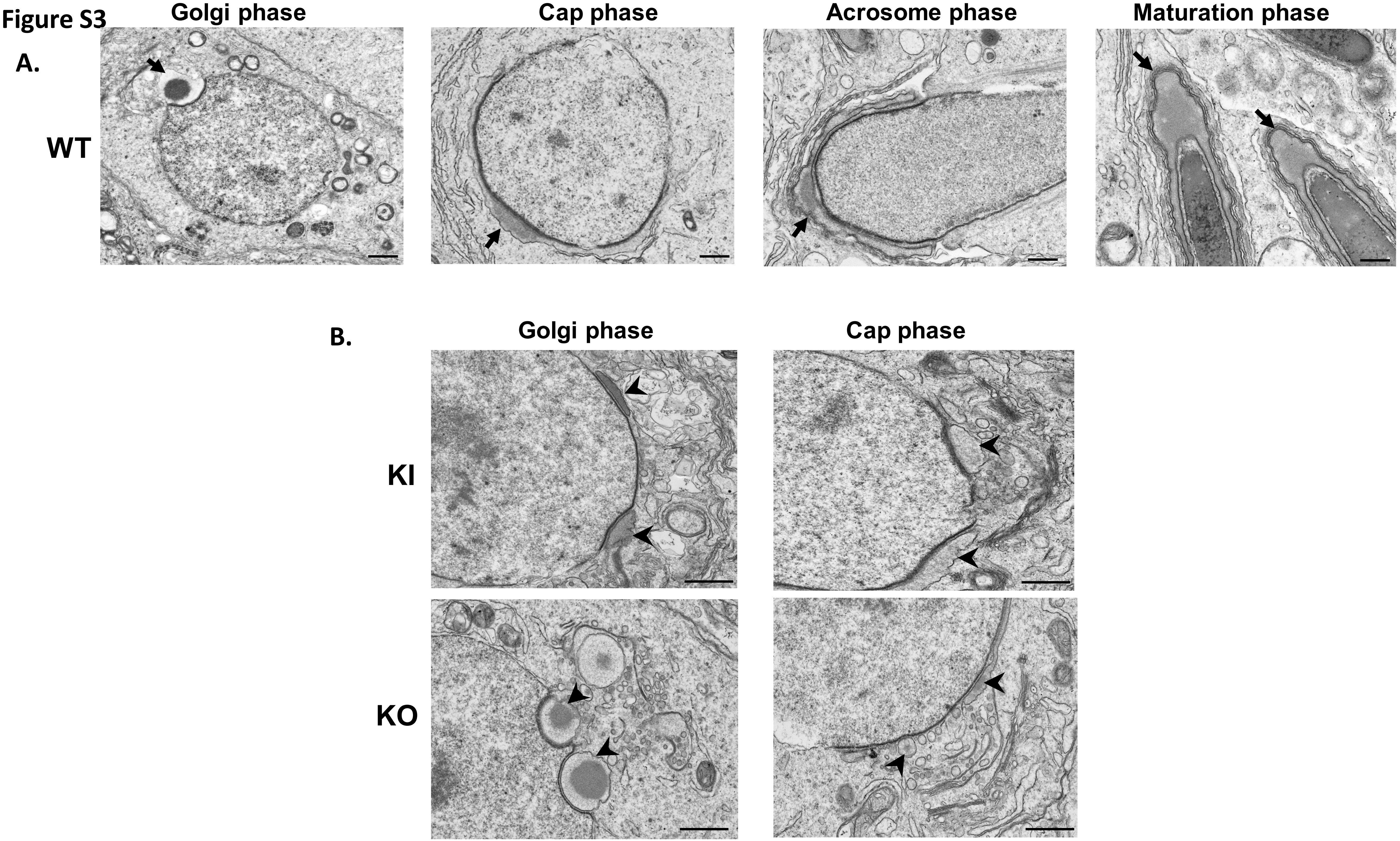
A.
Golgi phase
Cap phase
Acrosome phase
Maturation phase
WT
B.
Golgi phase
Cap phase
KI
KO
